# An integrated active case detection and management of skin NTDs in yaws endemic health districts in Cameroon, Côte d’Ivoire and Ghana

**DOI:** 10.1371/journal.pntd.0011790

**Published:** 2024-10-04

**Authors:** Serges Tchatchouang, Laud A. Basing, Hugues Kouadio-Aboh, Becca L. Handley, Camila G-Beiras, Ivy Amanor, Philippe Ndzomo, Mohammed Bakheit, Lisa Becherer, Sascha Knauf, Claudia Müller, Earnest Njih-Tabah, Theophilus Njamnshi, Tania Crucitti, Nadine Borst, Simone Lüert, Sieghard Frischmann, Helena Gmoser, Emelie Landmann, Aboubacar Sylla, Mireille S. Kouamé-Sina, Daniel Arhinful, Patrick Awondo, Gely Menguena, Emma-Michèle Harding-Esch, Adingra Tano, Mamadou Kaloga, Paul Koffi-Aboa, Nana Konama-Kotey, Oriol Mitjà, Sara Eyangoh, Kennedy Kwasi-Addo, Solange Ngazoa-Kakou, Michael Marks

**Affiliations:** 1 Centre Pasteur du Cameroun, Yaoundé, Cameroon; 2 Noguchi Memorial Institute for Medical Research, University of Ghana, Legon, Accra, Ghana; 3 Institut Pasteur de Cote d’Ivoire, Abidjan, Lagunes, Côte d’Ivoire; 4 National Program of African Trypanosomiasis Elimination, Abidjan, Côte d’Ivoire; 5 Clinical Research Department, London School of Hygiene & Tropical Medicine, London, United Kingdom; 6 Skin Neglected Tropical Diseases and Sexually Transmitted Infections section, Hospital Universitari Germans Trías i Pujol; Fight Infectious Diseases Foundation,Badalona, Barcelona, Spain; 7 Universitat Autònoma de Barcelona, Bellaterra, Cerdanyola del Vallès, Barcelona, Spain; 8 Mast Diagnostica GmbH, Reinfeld, Germany; 9 Laboratory for MEMS Applications, IMTEK—Department of Microsystems Engineering, University of Freiburg, Freiburg, Germany; 10 Institute of International Animal Health/One Health, Friedrich-Loeffler-Institut, Greifswald—Insel Riems, Germany; 11 Professorship for One Health/International Animal Health, Faculty of Veterinary Medicine, Justus Liebig University, Giessen, Germany; 12 National Buruli Ulcer, Leprosy, Yaws and Leishmaniasis Control Program, Ministry of Public Health, Yaounde, Centre Region, Cameroon; 13 Public Health & Epidemiology, University of Dschang, Dschang, West Region, Cameroon; 14 Experimental Bacteriology Unit, Institut Pasteur de Madagascar, Antananarivo, Madagascar; 15 Hahn-Schickard, Freiburg, Germany; 16 Clinical Research Department, London School of Hygiene & Tropical Medicine, Faculty of Infectious and Tropical Diseases, London, United Kingdom; 17 Programme National de Lutte contre l’Ulcère de Buruli, Abidjan, Côte d’Ivoire; 18 National Yaws Eradication Program, Ghana Health Service, Accra, Ghana; 19 Hospital for Tropical Diseases, London, United Kingdom; University of California, Los Angeles, UNITED STATES OF AMERICA

## Abstract

**Background:**

Integrated approaches to mapping skin Neglected Tropical Diseases (NTDs) may be cost-effective way to guide decisions on resource mobilization. Pilot studies have been carried out, but large-scale data covering multiple countries endemic for skin NTDs are lacking. Within the LAMP4YAWS project, we collected integrated data on the burden of multiple skin NTDs.

**Methods:**

From March 2021 to March 2023, integrated case searches for yaws alongside other skin conditions were performed in endemic health districts of yaws in Cameroon, Côte d’Ivoire, and Ghana. Integrated activities included training, social mobilization and active case detection. Initial screening involved a brief clinical examination of participants to determine if any skin conditions were suspected. Cases of skin NTDs were then referred to a health facility for appropriate management.

**Results:**

Overall 61,080 individuals screened, 11,387 (18.6%) had skin lesions. The majority of individuals (>90%) examined were children aged 15 years old and under. The proportion of serologically confirmed yaws cases was 8.6% (18/210) in Cameroon, 6.8% (84/1232) in Côte d’Ivoire, and 26.8% (440/1643) in Ghana. Other skin conditions based on clinical examination included: scabies, Buruli ulcer, leprosy, lymphatic filariasis (lymphoedema and hydrocele), tungiasis, and fungal infections. The most common conditions were scabies and superficial fungal infections. In Cameroon, scabies and superficial fungal infections accounted for 5.1% (214/4204) and 88.7% (3730/4204) respectively, 25.2% (1285/5095) and 50.4% (2567/5095) in Côte d’Ivoire. In Ghana, 20% (419/2090) of individuals had scabies but superficial fungal infections were not routinely recorded and were reported in only 1.3% (28/2090). Other skin NTDs were less common across all three countries.

**Conclusion:**

This study confirms that integrated screening allows simultaneous detection of multiple skin NTDs, maximising use of scarce resources.

## Author summary

Many Neglected Tropical Diseases (NTDs) predominantly affect the skin and are referred to as skin NTDs. The World Health Organization (WHO) has developed a number of strategies for the control, eradication and elimination of skin NTDs and recognizes the importance of integrated approaches to mapping skin NTDs. We conducted a study adopting integrated screening for multiple skin NTDs and other skin conditions in Cameroon, Côte d’Ivoire, and Ghana. This ran alongside a study focused on diagnostic tests for one specific skin NTD–yaws.

The results showed that integrated screening is a feasible and cost-effective way to detect multiple skin NTDs in a single intervention. Of more than 60,000 individuals screened almost one in five had a skin lesion. Cases of yaws confirmed by blood tests were detected more frequently in Ghana compared to Côte d’Ivoire and Cameroon. The most common skin conditions were scabies and superficial fungal infections. Other skin NTDs such as Buruli ulcer, leprosy, lymphatic filariasis and tungiasis were less common. Integrated screening allowed detection of skin conditions and co-endemicity of skin NTDs and the data can guide decisions on resource mobilization to manage skin NTDs.

## Introduction

Skin diseases are a significant global health issue which affect almost 900 million people in the world. They represent the third most common reason for seeking medical care [1–3]. A group of these conditions are classified by the World Health Organization (WHO) as Neglected Tropical Diseases (NTDs) [4]. Not all NTDs are dermatological conditions, but there are ten NTDs that specifically manifest as skin diseases (skin NTDs). If not diagnosed and treated promptly, these conditions can result in disfigurement, disability, stigmatisation, impacts on mental health, and socioeconomic difficulties [4–6].

In Cameroon, Côte d’Ivoire, and Ghana, several skin NTDs are known to be endemic including yaws [7–10], Buruli ulcer [11–13], leishmaniasis [14–16], leprosy [10,17,18], lymphatic filariasis [19–21], mycetoma [22–24], onchocerciasis [25–27], and scabies [10,28–30]. Alongside these NTDs, a number of more common skin conditions including superficial fungal and bacterial infections are known to cause a considerable burden of disease [28,31–33].

Most data on skin NTDs rely on passive surveillance activities, which are likely to underestimate the true burden of disease as they are dependent on patients who present to a health centre, receive a diagnosis and are reported to the respective disease control programme. The highest burden of skin NTDs are believed to be in communities living in difficult to reach areas with limited access to healthcare [34,35]. Consequently, routine data are likely to underreport the true burden and epidemiology of these conditions.

To achieve the WHO goals for skin NTDs, active case detection will be required. Therefore, WHO has recently promoted integrated activities of skin NTDs in order to accelerate progress in their control, elimination and eradication [4,36]. Because NTDs often overlap in geographic areas, integrated active case searching may offer a way to overcome some of the challenges associated with stand-alone programmes and reduce the risk of late detection of skin NTDs. Integration can also result in a reduction of resources, thereby increasing cost-effectiveness and allow for an expanded coverage of interventions [2,36–38].

The LAMP4YAWS project in Cameroon, Côte d’Ivoire, and Ghana aimed to implement and evaluate a loop-mediated isothermal amplification test for yaws. As part of the project, field activities included active case searching of yaws using an integrated approach [39]. Here we report data on skin conditions from active case searches in yaws-endemic health districts from all three countries.

## Methods

### Ethics statement

This study was approved in Cameroon by the National Ethics Committee on Research for Human Health (CNERSH) under decision N°2020/12/1327/CE/CNERSH/SP and administrative authorization of the Ministry of Public Health N°D30-308/L/MINSANTE/SG/DROS), in Côte d’Ivoire by the Comité National d’Ethique des Sciences de la Vie et de la Santé (IQR) through decision IQRG0075_16/09/2020, in Ghana by Ethical Review Committee of the Ghana Health Service (GHS-ERC) and Noguchi Memorial Institute for Medical Research-Institutional Review Board (NMIMR-IRB) under decisions GHS-ERC 005/12/20 and NMIMR-IRB CPS 019/20-21), and in United Kingdom through London School of Hygiene and Tropical Medicine (LSHTM) under decision N° 21633 (19 August 2021).

All participants and/or the parents/legal guardians of minors provided written informed consent before enrolment. In addition, assent was obtained from child participants.

### Study design

From March 2021 to March 2023, we conducted integrated case searches in rural communities within suspected yaws-endemic health districts in Cameroon, Côte d’Ivoire, and Ghana. Integrated approach for skin NTDs offers possibility to address several diseases simultaneously in the same communities in order to maximize the utilization of limited resources [40]. This approach was implemented by assessing data on yaws in health districts from notifications of NTD national programmes, training of health workers and village volunteers, conducting active case detection of skin conditions, clinical and serological diagnosis of yaws, treating yaws cases and referring other skin conditions to healthcare centres [2]. In each community visited, the initial screening involved an examination to determine if participants had yaws-like lesions as described in the study protocol or other skin conditions [39].

### Study sites

The study was conducted in rural areas in 12 health districts in Cameroon, six in Côte d’Ivoire, and seven in Ghana [39]. The characteristics of the study sites are summarised in Table 1. Study sites were selected based on clinical notifications of yaws by the national programmes on NTDs.

**Table 1:** Characteristic of study sites in Cameroon, Côte d’Ivoire and Ghana.

Country	Region	Health district	Sub-divisions	Population	Active case searching strategy
Cameroon	East	Doumé	Doumé, Doumaitang, Dimako	53,286	Community, House-to-house
		Bétaré-Oya	Betaré-Oya, Ngoura	141,405	Community, health centres
		Batouri	Batouri, Ndemnam	121,199	Community, health centres
		Messamena	Messamena, Somalomo	48,000	Community, health centres
		Abong-Mbang	Abong-Mbang, Atok, Angossas, Dja (Mindourou)	85,741	Community, health centres
		Lomié	Lomié, Messok, Ngoyla	47,695	Community, health centres
		Yokadouma	Gari-Gombo, Yokadouma	126,353	Community, health centres
		Mbang	Mbang	34,148	Community, House-to-house
	South	Djoum	Djoum, Oveng, Mintom	43,142	MDA
		Lolodorf	Bipindi, Lolodorf, Mvengue, Akom 2	43,446	Community
		Sangmélima	Sangmelima, Meyomessi	110,385	School, community
	Adamawa	Bankim	Bankim	121,756	School, community, church
Côte d’Ivoire	Loh Djiboua	Divo	Goh-Djiboua	458,318	School, Community
	Agneby Tiassa	Tiassalé	Lagunes	270,412	
	Bélier	Toumodi	Lacs	189,794	
		Autonomous district of Yamoussoukro	N/A	446,158	
	Haut sassandra	Daloa	Sassandra-Marahou	745,628	
		Vavoua	Sassandra-Marahou	451,492	
Ghana	Central	Asikuma Odoben Brakwa	N/A	129,580	School, Community
		Ajumako Enyan Essiam	N/A	138,046	
	Eastern	Upper West Akim	N/A	93,391	
		Ayensuano	N/A	94,594	
		Akuapim North	N/A	105,315	
		Asene Manso Akroso	N/A	77,498	
	Western	Mpohor	N/A	52,473	

MDA: Mass drug administration; N/A: not applicable

### Recruitment of participants

Case searches were conducted in collaboration with national NTD programmes. District health staff were first trained to recognise skin NTDs as well as other skin conditions. Training materials included the WHO manual on recognition of skin NTDs [41], as well as other national NTD training materials.

Individuals presenting with yaws-like lesions underwent SD Bioline (Abbott, USA) test to detect anti-treponemal antibodies. Those reactive underwent a Chembio Dual Path Platform (DPP) test (Chembio Diagnostics, New York, USA) for simultaneous detection of both treponemal and non-treponemal antibodies, interpreted as serologically confirmed yaws case. Samples were then taken as part of the main study from participants as described elsewhere [39]. Other skin diseases were recorded and referred to the health district for management following the national guidelines.

Several approaches were used for case finding, including community-based case detection, detection in healthcare centres, primary school-based screening programs, screening alongside mass drug administration (MDA) campaigns, church and house-to-house case searches (Table 1). The strategy was chosen based on other activities taking place in the selected district. The research team visited different study sites according to a pre-established schedule that was communicated to health workers, village chiefs, and school directors before the appointed day.

### Case definition of other skin conditions

In order to be considered a case of leprosy (Hansen’s Disease), an individual must have had one or more skin lesion that was hypopigmented or red and had definite loss of sensation.

Buruli ulcer case was based on clinical presentation consistent with a painless, non-healing ulcer with undermined edges and a necrotic base, typically located on the limbs; a painless nodule or plaque, typically located on the limbs; and oedema of a limb or other body part, with or without overlying ulceration.

Scabies were defined as people with rash consisting of papules, vesicles, and burrows, typically located on the hands, feet, and folds of the skin.

An individual suffering from lymphatic filariasis presented lymphedema characterised by the enlargement of body parts in the limbs and breasts; or hydrocele marked by swelling in the scrotal sack.

Case of tungiasis was defined as participant with a painful, pruritic nodule with a central black dot, mainly located on the feet, toes, or fingers.

Superficial fungal infections were defined as an affection on the body commonly with a red, ring-shaped rash with a raised border for *Tinea corporis* (ringworm); scaly patches on the scalp sometime with hair loss for *Tinea capitis* (scalp ringworm); light or dark patches on the skin, most commonly on the chest and back for *Tinea versicolor* (pityriasis versicolor).

### Data analyses

Study data were recorded using standardized data collection forms. We used basic descriptive statistics to summarise the characteristics of the individuals who were screened, the number and proportion who were diagnosed with each skin NTD, and other skin conditions. Results are shown stratified by country. Analyses were performed in Microsoft Excel 2013.

## Results

### Demographic characteristics

Case searches were conducted in 12 districts in Cameroon, six in Cote d’Ivoire, and seven in Ghana. A total of 61,080 individuals were screened, including 20,414 in Cameroon, 16,530 in Côte d’Ivoire, and 24,136 in Ghana. The male to female ratio was approximately 1:1 in the three countries (10804/9610, 8430/8100, and 13241/10895 respectively for Cameroon, Côte d’Ivoire and Ghana), with more than 90% of participants aged up to 15 years old (56471/61080).

### Skin lesions detected

Table 2 summarizes the skin diseases detected in Cameroon, Côte d’Ivoire, and Ghana. Skin lesion accounted for 18.6% (11389 /61080) and included 20.6% (4204/20414) in Cameroon, 30.8% (5095/16530) in Côte d’Ivoire and 8.7% (2090/24136) in Ghana (see supporting information: S1, S2 and S3 Data and S1 Text).

**Table 2:** Characteristics of skin diseases found in Cameroon, Côte d’Ivoire and Ghana.

Country	Health District	Population screened	Leprosy	Scabies	Lymphoedema	Hydrocele	*Pityriasis versicolor*	*Tinea capitis*	*Tinea corporis*	Suspected cases of BU	Suspected cases of yaws	Sd positive	DPP positive
Cameroon	Abong-Mbang	223	0	3	0	0	0	16	0	2	7	0	0
	Bankim	6911	2	81	0	0	112	1552	67	0	27	0	0
	Batouri	268	0	0	0	0	0	3	1	0	0	0	0
	Bétaré-Oya	1281	5	3	0	8	0	121	9	0	6	0	0
	Djoum	1344	0	7	1	0	0	241	1	0	47	1	1
	Doumé	1141	6	2	0	0	22	238	0	2	7	0	0
	Lolodorf	1102	0	10	0	0	4	102	1	1	5	0	0
	Lomié	1868	0	5	3	0	0	119	0	1	11	3	2
	Mbang	976	0	21	0	2	25	199	5	0	33	19	15
	Messamena	814	1	27	0	2	0	97	0	7	4	0	0
	Sangmélima	3150	0	51	2	0	0	580	0	0	49	0	0
	Yokadouma	1336	1	4	0	1	5	210	0	1	14	1	0
	**Sub-total**	**20414**	**15**	**214**	**6**	**13**	**168**	**3478**	**84**	**14**	**210**	**24**	**18**
Côte d’Ivoire	Divo	3080	1	180	0	0	134	187	39	2	112	12	11
	Tiassalé	4830	0	430	1	3	289	405	88	3	473	87	54
	Yamoussoukro	3700	0	221	0	0	144	201	42	0	259	16	12
	Toumodi	2160	0	132	0	0	115	161	34	0	151	5	4
	Daloa	2100	0	215	0	0	192	268	57	0	191	7	3
	Vavoua	660	1	107	0	0	86	120	25	0	46	0	0
	**Sub-total**	**16530**	**2**	**1285**	**1**	**3**	**960**	**1342**	**285**	**5**	**1 232**	**127**	**84**
Ghana	Ayensuanor	3119	0	11	0	0	0	0	0	0	194	29	23
	Asikuma Odoben Brakwa	3496	0	0	0	0	0	0	0	0	201	56	43
	Asene Manso Akroso	3331	0	86	0	0	0	14	0	0	306	204	196
	Akuapim North	3817	0	14	0	0	0	3	0	0	201	27	16
	Mpohor	3323	0	86	0	0	0	1	0	0	206	80	74
	Ajumako Enyan Essiam	2864	0	98	0	0	0	2	0	0	211	15	8
	Upper West Akim	4186	0	124	0	0	0	8	0	0	324	89	80
	**Sub-total**	**24136**	**0**	**419**	**0**	**0**	**0**	**28**	**0**	**0**	**1643**	**500**	**440**

DPP+: Positive Dual Path Platform

The most frequent skin conditions encountered in Cameroon were *Tinea capitis*, scabies and yaws-like lesions. *T*. *capitis* accounted for 82.7% (3478/4204) of the skin conditions and was detected mainly in the health districts of Bankim (44.6%, 1552/3478)) and Sangmélima (16.7%, 580/3478)). Scabies was the second most common skin condition detected in 5.1% (214/4204) cases of skin conditions seen. The majority of scabies cases were again detected in Bankim and Sangmélima with 37.9% (81/214) and 23.8% (51/214) of scabies among the detected skin lesions, respectively. Yaws-like lesions were the third most common skin condition (5%, 210/4204) and were identified in high numbers in Sangmélima (23.3%, 49/210), Djoum (22.4%, 47/210) and Mbang (15.7%, 33/210). However, only 18 individuals were serologically confirmed as yaws cases almost all among Baka population.

*Pityriasis versicolor*, scabies, and yaws-like lesions accounted for 99.8% (5084/5095) of skin conditions detected in Côte d’Ivoire. *P*. *versicolor* was the most common skin condition (50.4% (2567/5095)) across the six health districts visited. Scabies represented 25.2% (1285/5095) of the conditions and yaws-like lesions were reported among 24.2% of individuals presenting with a skin condition (1232/5095) and were serologically confirmed in 84 people.

In Ghana, superficial fungal infections were not routinely recorded yaws-like lesions accounted for 78.6% (1643/2090) of suspected skin-NTD cases, with serologically confirmed yaws among 440 individuals. Upper West Akim District and Asene Manso Akroso recorded the highest number of yaws cases. Scabies and *T*. *capitis* accounted for 20% (419/2090) of skin conditions seen.

## Discussion

In accordance with the NTD road map 2021–2030 [4], skin NTDs are targeted for eradication (yaws), elimination (leprosy, onchocerciasis, lymphatic filariasis) and control (other skin NTDs), and an integrated approach is the backbone to achieving these targets. The main study was focused on yaws while adopting an integrated approach in the screening of individuals in the selected health districts.

In Cameroon, Côte d’Ivoire, and Ghana, among suspected cases, the frequency of serologically confirmed yaws detection was 8.6%, 6.8%, and 26.8%, respectively. These findings are in line with routine reporting data in Ghana that indicates frequencies above 15% among suspected cases of yaws. [34,42,43]. Ghana has the second-highest population density in West Africa, which may make containing the spread of yaws more challenging. For example, despite numerous MDA campaigns in the past, the incidence of yaws remains high in many regions of the nation [9,42,44]. The data obtained in Cameroon overlap with previous findings, with less than 10% of suspected yaws cases being serologically confirmed [45,46] but lower to what has been reported during an outbreak [8]. The decline can be associated to impact of surveillance in identifying and treating cases, as well as the implementation of MDA in the Congo Basin in December 2020 prior to the current study’s conduct. [47]. The results of Côte d’Ivoire show that yaws remains prevalent in the study sites compared to previous findings that identified 8 cases of yaws out of 1,302 individuals with skin conditions despite a number of studies and treatments that have been conducted [10]. Côte d’Ivoire is one of the countries in Africa that has been most affected by skin NTDs, including yaws [48,49]. [10]. The study’s findings indicate that more work is required to control yaws in the three nations, specifically in regards toMDA rounds that can be reviewed in order to considerably lower the disease’s incidence. [50]. In order to improve patient outcomes, it is important to properly identify and manage other aetiologies of cutaneous ulcers, such as *H*. *ducreyi* which has been shown to be present in as many as 80% of yaws-like ulcers. [51].

Scabies was the most common skin NTD detected including 25.2% in Côte d’Ivoire, 19.8% in Ghana and 5.1% in Cameroon. These data are high compared to previous studies in the three countries where proportions of scabies in people with skin lesions ranged almost from 3 to 10% during active case search. For instance, around 3% of scabies have been reported in rural settings in Cameroon a decade ago [28]. In Côte d’Ivoire, the proportions of scabies varied between 3.7% and 8.6% in earlier active case searching studies [10,48]. For Ghana, approximately 10% of cases of scabies were reported among high school students in Accra which may not be comparable to a rural setting [52]. Difference may be due to a number of factors, including the duration of the screening, the context of the study (i.e. hospital, prison or boarding school and outbreaks) and the methods used to detect scabies [30,53–57].

Other skin NTDs such as leprosy, Buruli ulcer, lymphatic filariasis and tungiasis were less common. Leprosy cases were only found in Cameroon and Côte d’Ivoire. In Ghana it is possible advancement in leprosy control meant no cases were present. All three countries are known to be endemic [11–13,19–21], however no cases were detected in Ghana. Tungiasis was only documented in Cameroon.

The study also found that superficial fungal infections were the most common form of skin disease in two of the three countries. In Cameroon, they accounted for almost 90% with the majority due to *Tinea capitis* while *Pityriasis versicolor* constituted the bulk of these infections in Côte d’Ivoire (50.4%). The findings are concordant with field data in Cameroon, Côte d’Ivoire and Togo highlighting fungal infections as the main skin conditions [10,28,31,58]. In previous investigation, proportion of *Pityriasis versicolor* and *Tinea capitis* among diagnosed skin diseases in Côte d’Ivoire was approximately 45% of each [31]; while in Cameroon proportion of *Pityriasis versicolor* was around 21% [28]. In Ghana, superficial fungal infections were likely to be less common but earlier data indicated that they accounted for between 20 and 30% of patient’s skin diseases [59,60]. The difference could be due to the teams in Ghana not automatically recording information on all superficial fungal infections.

This was an opportunistic study to record the presence of skin NTDs and other skin conditions whilst active case searching for yaws as part of the LAMP4YAWS project. This allowed us to utilise resources of an existing study to determine the prevalence of multiple conditions in the selected communities. As well as integrated mapping of similar conditions, integrated approaches can be effective in reducing the prevalence of NTDs through combined management, thus improving the health of people in these countries. Moreover, since many skin NTDs are zoonotic or have a (possible) non-human reservoir, a One Health guided approach should be used to handle integrated case search activities [61]. Synergies between skin NTD programmes and, for example, neglected zoonotic disease programmes (such as rabies elimination) could optimize the use of resources (logistics) and stimulate cross-sectoral collaboration that is key for the sustainable control of NTDs.

This study has some limitations. Since the diagnosis of the skin NTDs other than yaws was based on clinical examinations, the other reported cases were only suspected cases. Ghana did not systematically record data on superficial fungal infections. The information gathered, however, is still helpful in determining future directions for work related to skin NTD eradication, elimination, or control, including data collection, training, diagnostics, and the need for medical care in these isolated areas. As the case searches were designed to support evaluation of a diagnostic test for yaws, our activities were conducted in yaws endemic districts. It is possible that other skin NTDs may have been more common if we had conducted case detection activities elsewhere.

The lessons learned from this study include: the potential efficiency of the integrated approach in reporting various skin NTDs within the project’s framework and accomplishing the study’s objectives with the use of existing disease-specific resources; the challenges encountered such as hard-to-reach population, intercommunity movement and how to integrate management of the high burden other skin diseases alongside skin NTD activities; and the future research recommendations that could consider how affected populations move around due to rural activities like gathering and hunting, as well as what these communities’ basic healthcare needs are.

## Conclusion

We demonstrate improved data reporting of skin NTDs when an integrated approach is used. With the help of this study, the Yaws surveillance system is strengthened and made into a guided, integrated system that is implemented nationwide. The detection of yaws cases in areas that have undergone MDA is remarkable and highlights the importance of rigorous active surveillance ideally with test confirmation of clinically suspected cases and optimal coverage of MDA campaigns. The main challenges remain the difficulty of accessing some communities where yaws is endemic, the follow-up of patients, and the inter-community movements which are sources of reinfection, and resources to manage the affected populations.

## Supporting information

S1 Text List of legends for supporting information files. (DOCX)

S1 Data Cameroon data. (XLSX)

S2 Data Côte d’Ivoire data. (XLSX)

S3 Data Ghana data. (XLSX)
