# Supplementary material for: An integrated active case detection and management of skin NTDs in yaws endemic health districts in Cameroon, Côte d’Ivoire and Ghana
Source: PLoS Negl Trop Dis. 2024 Oct 4;18(10):e0011790. doi: 10.1371/journal.pntd.0011790 (PMC11482705; doi:10.1371/journal.pntd.0011790)
Supplement: S1 Text — (DOCX) [file pntd.0011790.s001.docx]

**Legend for the supporting files**

**Translation in S1 et S2 data**

| **French** | **English** |
| --- | --- |
| Sexe | Sex |
| Tranche d'age | Age group |
| Population | Population |
| Age-MTN | NTDs following age group |

**Definition of age groups**

| 1-11 Months | 1-4 years | 5-9 years | 10-15 years | over 15 years old |
| --- | --- | --- | --- | --- |
| 1 | 2 | 3 | 4 | 5 |

| **Age_ skin NTD** | <15 ans | 15 years old and above |
| --- | --- | --- |
|  | A | B |

**Meaning of abbreviations**

| BU | Buruli ulcer |
| --- | --- |
| DPP | Dual Path Platform |
| F | Female |
| M | Male |
| Neg | Negative |
| Pos | Positive |
